# GC-MS Profiling and Biomedical Applications of Essential Oil of *Euphorbia larica* Boiss.: A New Report

**DOI:** 10.3390/antiox12030662

**Published:** 2023-03-07

**Authors:** Muddaser Shah, Faizullah Khan, Saeed Ullah, Tapan Kumar Mohanta, Ajmal Khan, Rimsha Zainab, Naseem Rafiq, Hussan Ara, Tanveer Alam, Najeeb Ur Rehman, Ahmed Al-Harrasi

**Affiliations:** 1 Natural and Medical Sciences Research Center, University of Nizwa, PC 616, Birkat Al Mauz, Nizwa P.O. Box 33, Oman; 2 Department of Botany, Abdul Wali Khan University Mardan, Mardan 23200, Pakistan; 3 Department of Pharmacy, Abdul Wali Khan University Mardan, Mardan 23200, Pakistan; 4 Department of Botany, Women University Swabi, Swabi 23430, Pakistan; 5 Department of Zoology, Abdul Wali Khan University Mardan, Mardan 23200, Pakistan

**Keywords:** *Euphorbia larica* Boiss., antidiabetic, essential oil, GC-MS, cytotoxic, CA-II, antimicrobial, antioxidant, analgesic, anti-inflammatory

## Abstract

The present study explored *Euphorbia larica* essential oil (ELEO) constituents for the first time, obtained via hydro-distillation by means of Gas Chromatography-Mass Spectrometry (GC-MS) profiling. The essential oil was screened in vitro against breast cancer cells, normal cell lines, α-glucosidase, carbonic anhydrase-II (CA-II), free radical scavenging and in vivo analgesic and anti-inflammatory capabilities. The GC-MS screening revealed that the ELEO comprises sixty compounds (95.25%) with the dominant constituents being camphene (16.41%), thunbergol (15.33%), limonene (4.29%), eremophilene (3.77%), and β-eudesmol (3.51%). A promising antidiabetic capacity was noticed with an IC_50_ of 9.63 ± 0.22 μg/mL by the ELEO as equated to acarbose with an IC_50_ = 377.71 ± 1.34 μg/mL, while a 162.82 ± 1.24 μg/mL inhibition was observed against CA-II. Regarding breast cancer, the ELEO offered considerable cytotoxic capabilities against the triple-negative breast cancer (MDA-MB-231) cell lines, having an IC_50_ = 183.8 ± 1.6 μg/mL. Furthermore, the ELEO was also tested with the human breast epithelial (MCF-10A) cell line, and the findings also presumed that the ELEO did not produce any damage to the tested normal cell lines. The ELEO was effective against the Gram-positive bacteria and offered a 19.8 ± 0.02 mm zone of inhibition (ZOI) against *B. atrophaeus*. At the same time, the maximum resistance with 18.03 ± 0.01 mm ZOI against the fungal strain *Aspergillus parasiticus* was observed among the tested fungal strains. An appreciable free radical significance was observed via the DPPH assay with an IC_50_ = 133.53 ± 0.19 µg/mL as equated to the ABTS assay having an IC_50_ = 154.93 ± 0.17 µg/mL. The ELEO also offered a substantial analgesic capacity and produced 58.33% inhibition in comparison with aspirin, a 68.47% decrease in writhes, and an anti-inflammatory capability of 65.54% inhibition, as equated to the standard diclofenac sodium having 73.64% inhibition. Hence, it was concluded that the ELEO might be a natural source for the treatment of diabetes mellitus, breast cancer, analgesic, inflammatory, and antimicrobial-related diseases. Moreover, additional phytochemical and pharmacological studies are needed to isolate responsible chemical ingredients to formulate new drugs for the examined activities.

## 1. Introduction

Medicinal plants are the prime source of alternative therapy for diabetes mellitus (DM) and numerous other human health complications [1]. Essential oils (EOs) produced from plants have multiple pharmacological and pharmaceutical properties including microbial resistance [1,2,3], scavenging free radicals [2,3], curing inflammation [4,5,6], α-glucosidase inhibition [7], carbonic anhydrase-II inhibition [8,9], and relieving pain [10]. In recent eras, the EOs obtained from medicinal herbs have gained attention due to their diverse biomedical applications including antidiabetic, antifungal, antibacterial, and pharmacological capabilities, as well as effective natural remedies [7,8]. Furthermore, essential oils are widely utilized over the globe with less adverse effects, easy availability, affordability, and a high rate of efficacy [11,12], which have attracted considerable interest [13].

Cancer is an intricate genetic disease that retards the further growth and expansion of normal cells in the living body, even leading to death [14]. According to recent statistics (2020), cancer has been ranked second most common among the main causes of mortality over the globe, and around 9.6 million people died from cancer in 2018 [15]. The exploration of new medications using therapeutic plants that can heal cancer has become one of the most exhilarating demands through phytochemical screening [16]. However, medicinal plants have offered a significant role in developing traditional medicine systems, particularly to treat cancer [17]. In addition, EO constituents have also played an important role in cancer prevention and treatment [13]. In recent decades, most of the plants bearing essential oil displayed anticancer [13,14,18] and antitumor [19] activities to overcome the expansion of multidrug resistance complications [20]. Because of the considerable capabilities of the essential oils in cytotoxic treatment, the ELEO could be used as a complementary therapy to natural and chemotherapeutic drugs [21].

Carbonic anhydrases (CAs) are metalloenzymes that frequently persist in living organisms and catalyze the transformation of CO_2_ and H_2_O to HCO^3−^ and H^+^ [22]. There are 16 different CA isozymes [23], and CAs play a vigorous role in mammals in several processes such as pH control, ion transport, calcification, stability, and secretion of electrolytes [22]. Classical CA (acetazolamide and brinzolamide) inhibitors have been used as commercial medications in the healing of numerous ailments comprising edema, cancer, glaucoma, epilepsy, obesity, and osteoporosis [24] for a long time with undesired side effects [25,26]. Thus, searching for new natural and safer CA inhibitors with fewer side effects is required.

A deficiency of insulin secretion usually causes type I diabetes (T1DM) [27]. Type I diabetes (T1DM) is typically instigated by the deficiency of insulin secretion. In contrast, type II diabetes (T2DM) is instigated by increased insulin resistance in the liver, decreased cell mass of peripheral organs, and insufficient insulin production [28]. Approximately 90% of diabetic people have T2DM [29]. Furthermore, α-glucosidase (AGIs) inhibitors are efficient and perform a substantial role in reducing post-prandial glucose levels. The α-Glucosidase enzyme (EC 3.2.1.20) catalyzes the release of α-glucosides from the non-reducing side of the carbohydrates, consequently inhibiting the enzyme to control the raised glucose levels in the human blood [30]. α-Glucosidase (AGIs) inhibitors are effective and play a significant role in reducing post-prandial glucose levels. Numerous side effects, such as diarrhea and abdominal pain, are due to the regular use of synthetic drugs [17,18,19]. Hence, developing new natural AGIs with high efficiency is highly essential without imposing greater side effects.

*Euphorbia* is the largest genus, comprising around 2040 species in the family Euphorbiaceae [31]. Some plant species of *Euphorbia* have been documented for various health complications such as skin diseases, migraine, gonorrhea, intestinal parasites, and warts [32]. *Euphorbia larica* Boiss (Local name = Isbaq) is a significant native medicinal plant, mostly distributed over stony and rocky (up to one-meter-high) places of Dhofar (Salalah) and Ad Dakhiliyah areas of Oman. The native individuals of Oman habitually use sap or latex of *E. larica* to patch bites, burn wounds [33], as well as for treating camels with parasites [34]. Quercetin and kaempferol derivatives such as kaempferol-3-rutinoside, kaempferol-3-O-glucoside, quercetin 3-O-glucoside, rutin, and 6-methoxyapigenin were observed in ethanolic extracts of *E. larica* leaf [35]. In addition, saturated and oxygenated hydrocarbons and esters were identified and isolated from the aerial parts of *E. larica* [36]. Recently, an anthracene derivative (eupholaricanone) with α-glucosidase inhibition along with three steroids was reported from aerial parts of *E. larica* [28]. Hence, to highlight the significance of the plant essential oil under study, for the first time GC-MS profiling, α-glucosidase inhibition, and antioxidant, antimicrobial, analgesic, anti-inflammatory, and breast cancer activities are available in the literature. Therefore, our main aim was to examine and evaluate the chemical arrangement and biological capabilities of ELEO as a first step in evaluating the prospective benefits of the plant species.

## 2. Materials and Methods

### 2.1. Plant Material and Identification

The whole aerial fresh plant parts of *E. larica* (3.5 Kg) were gathered from different regions of the Ad Dakhiliyah in the Sultanate of Oman. The plant under study was identified by Dr. Syed Abdullah Gilani at the Department of Biological Sciences and Chemistry, College of Arts and Sciences, University of Nizwa, Oman. After identification, the sample was cleaned, washed with distilled water, air-dried, and then grinded into a fine powder via a stainless-steel blender. The voucher specimen of *E. larica* (EL/03/2019) was kept in the herbarium at the Natural and Medical Sciences Research Center (NMSRC), University of Nizwa (UON), Nizwa, Oman.

### 2.2. Extraction of EO

The hydro-distillation of fresh aerial parts (EL, 0.98 g, 0.32%) of the *E. larica* growing in Oman gives yellow-colored EO (3 × 8 h) via Clevenger-type apparatus [7,37]. ELEO was obtained from the burette, dried over anhydrous sodium sulphate (Na_2_SO_4_), then weighed and preserved in the fridge at 4 °C until GC/MS profiling and biological screening.

### 2.3. GC/MS Profiling of the ELEO

The GC-MS screening of ELEO was performed via GC-MS. The GC-MS apparatus comprises the Perkin Elmer Clarus (PEC) 600 GC system connected with the Rtx-5MS capillary column (30 m × 0.25 mm I.D × 0.25 µm film thickness; the extreme temperature of 260 °C, fixed to a PEC 600 MS) (Waltham, MA, USA). The ultra-high-purity helium (He, 99.99%) was utilized as carrier gas having a flow frequency of 1.0 mL/min. Furthermore, the ionization energy (IE) was observed at 70 eV, and the electron multiplier voltage was adjusted from the auto-tune, whereas the injection, transfer line, and ion source temperatures were 260, 270, and 280 °C, respectively. The tested samples were 1 µL each, having a split ratio of 10:1. The oven was adjusted at 60 °C (for 1 min) at a rate of 4 °C/min—260 °C held for 4 min. The sample completed its run in 65 min. All the data of the ELEO were obtained by collecting the full scan mass spectra within 45–550 a.m.u. scan range.

### 2.4. Identification of Compounds

GC-MS determined the basic ingredients in the ELEO, and the unidentified compounds were identified and authenticated via MS library software (NIST 2011 v.2.3 and Wiley MS, 9th edition) and available literature. The chemical ingredients were quantified via an external standard via calibration curves generated through applying the GC screening of the representative compound groups. In addition to comparing RI (obs.) with RI (Lit.), the co-injection with an available authentic sample of detected chemical ingredients to GC or GC/MS was similarly used to identify the chemical ingredients.

### 2.5. In Vitro Cytotoxic Capacity

In vitro cytotoxicity capability of ELEO was evaluated using the MTT (yellow tetrazolium salt, 3-(4,5-dimethylthizol-2-yl)-2,5-diphenyl tetrazolium bromide) test utilizing breast cancer cell lines (MDA-MB-231) [38]. This study used the human breast normal cell line (MCF-10A) as a control. The MDA-MB-231 cell lines were acquired from the American Type Culture Collection (ATCC) and MCF-10A was purchased from the Iranian Biological Resource Center (IBRC) (Tehran, Iran). Cells were uniformly cultured in DMEM (Dulbecco’s Modified Eagle Medium) and further augmented together with 1% antibiotics (100 U/mL penicillin) and around 10% FBS. The cells were equally injected in a 96-well plate at 1.0 × 10^4^ cells/well density and then incubated for 24 h at 37 °C in 5% CO_2_. The medium was discarded, and both the cell lines were tested using ELEO at dosages of 3, 10, 30, 100, and 300 μg/mL, respectively [39]. Right after 48 h of the incubation [17], around 20 μL MTT solution, 5 mg/mL, was inoculated into every well and incubated for a further 4 h. However, the medium was then discarded, and the formazan precipitate was carefully homogenized in DMSO (dimethyl sulfoxide). The absorbances of the mixture’s reagents were examined via a microplate reader at 570 nm. All tests were carried out in triplicate, and the cytotoxicity potential was represented as a % of cell viability equated to untreated control cells following Equation (1) [39].

(1) $$\%\;\mathrm{viability}\;=\frac{OD_{test\;compound}}{OD_{control}}\times 100$$

where OD is the optical density of tested and controlled compounds.

### 2.6. In Vitro α-Glucosidase Potential

To evaluate the α-glucosidase inhibitory ability the ELEO was ensued at 37 °C using 0.5 mM phosphate buffer (pH 6.8) [7,30]. Furthermore, high to low dosages of the ELEO (60, 30, 15, 7.8, 3.90, and 1.95 µg/mL) were incubated through the α-glucosidase enzyme (2 U/2 mL) in phosphate buffer for 15 min at 37 °C. Later on, after the addition of 25 µL of substrate (p-nitrophenyl-a-D-glucopyranoside, 0.7 mM, final), the variations in absorbance at 400 nm for 30 min were noted using a microplate reader (Bio-Rad, Molecular Devices, CA, USA). DMSO-d (7.5 percent final) was applied as a positive control. In the current screening, acarbose with an IC_50_ 377.7 1.34 µg/mL was applied as a standard. The results were obtained by estimating IC_50_ through EZ-fit software via Equations (1) and (2).

(2) $$\;\;SE=\frac{\sigma }{\sqrt{n}}\;\;\;\;\;\;\;\;\;\;\;\;\;\;\;\;\;\;\;\;\;\;\;\;\;\;\;\;\;\;\;\;\;\;\;\;\;\;\;\;\;$$

### 2.7. In Vitro Carbonic Anhydrase-II Bioassay

The ELEO was screened for its CA-II inhibition significance using the most cited technique as stated by Rehman et al. [40]. To proceed with the assay, a freshly arranged aqueous solution of 20 bovine erythrocyte CA-II (0.1 mg/mL); 20 mL 4-Nitrophenyl acetate (4-NPA, 0.7 mM) in ethanol; and buffer solution (140 mL) HEPES-trisTris-HCl (20 mM) with pH 7.4 were properly homogenized in dimethyl sulfoxide (DMSO, 10%) in 96-well plates. The ELEO, standard, and enzyme (EC 4.2.1.1, Sigma-Aldrich, St. Louis, MO, USA) were carefully incubated for 15 min using a 96-well plate. Furthermore, the reaction proceeded by adding 20 μL 4-NPA and examining the rate of product formation for 30 min accurately with intervals of 1 min at 25 °C via a microplate reader. The significance was estimated through % inhibition using Equation (1).

### 2.8. Antimicrobial Assessment

Plants and their products are an alternative source of innovative drugs. Hence, to authenticate and highlight their importance, the *E. larica* essential oil was screened against the human pathogenic microbes using agar well diffusion techniques, and ZOIs were measured [41]. The microbial strains (clinical isolates) were identified by Dr. Hazir Rahman, Department of Microbiology, AWKUM, Mardan.

#### 2.8.1. Antibacterial Screening

The ELEO was screened against the bacterial strains *S. typhi*, *K. pneumonia*, *B. atrophaeus*, and *B. subtilis* via agar well diffusion (AWD) techniques [41,42]. For the antibacterial valuation, the media were arranged by adding 28 g of nutrient agar (NA) media along with 1 L of distilled water (DW) and shaken until the media was entirely homogenized. All the required apparatus was sterilized including prepared media, well borer, Petri plates, and wire loop, properly autoclaved at 121 ℃ for 15 min. Then, around 20 mL of nutrient agar autoclaved media was loaded into every Petri plate using a laminar flow hood and kept undisturbed until their solidification. The tested bacterial strains were carefully inoculated following safety measures using a wire loop at a concentration of bacterial cell density of 1.5 × 108 CFU/mL. Five wells of equal magnitude (3 mm) and the same distance were made over the solidified media using a sterilized corn borer.

Furthermore, around 1 mg from the ELEO and respective standards were dissolved in 1 mL of dimethyl sulfoxide (DMSO) to obtain a 1000 ppm stock solution from which 50 and 100 µL samples were taken, which were represented as 50 µg/mL and 100 µg/mL.

The ELEO at doses of 50 µg/mL was inoculated into the 1st well and 100 µg/mL was into the 2nd well, individually. Furthermore, the 3rd and 4th wells in the Petri plate were filled with standard levofloxacin and erythromycin at the same concentrations for Gram-positive and Gram-negative bacterial strains, correspondingly. At the same time, DMSO was added to the 5th well as a negative control over the nutrient agar media. The Petri plates were packed appropriately, labeled, and retained overnight in the incubator at 37 °C. Then, the glass Petri plates were carried out from the incubator, and resistance produced by the tested samples was observed as the zone of inhibition (ZOI) around the wells, determined in millimeters. All of the data were taken in triplicate, authenticated statistically, and expressed as mean ± SEM.

#### 2.8.2. Antifungal Evaluation

The ELEO was examined for its antifungal capabilities against *A. parasiticus* and *A. niger* from low to high doses via an agar well diffusion technique [41,42]. The antifungal activity was carried out by adding 39 g of the potato dextrose agar (PDA) to 1 L of distilled water in a glass conical flask and shaken until homogenized. Then, the antifungal media (PDA), Petri dishes, wire loop, and 3 mm steel borer were carefully autoclaved at 121 °C for around 20 min. The PDA media at about 20 mL was loaded into every glass Petri dish and afterward placed until media solidification. The tested fungal inoculum at a dosage of 108–109 CFU/mL was uniformly spread on the congealed PDA media. Later, around five wells using a borer of 3 mm were made at equal distances from one another. Low to high doses of ELEO were carefully inoculated into the 1st and 2nd wells over the media, and the standard fluconazole at the same doses was injected into the 3rd and 4th wells. At the same time, the 5th well was filled with DMSO, which was employed as a negative control. Next, all the Petri plates were carefully packed, labeled, and incubated for 72 h at 25 °C. Lastly, the Petri plates were brought out and the zone of inhibition was examined around the wells in millimeters. All of the data were taken in triplicate and expressed as mean ± SEM.

### 2.9. Antioxidant Screening

#### 2.9.1. DPPH Assay

The ELEO was examined for its antioxidant significance using a DPPH bioassay [41,43]. The antioxidant assay proceeded by homogenizing 3 mg DPPH with 100 mL of distilled methanol (DM). Then, the reagent was kept in the shade for around 30 min to produce the free radicals to appraise the antioxidant potential of the ELEO. The ELEO and ascorbic acid were prepared at dosages of 1000, 500, 250, 125, and 62.5 µg/mL to screen their antioxidant effects. Moreover, around 2 mL of ELEO and standard was added to 2 mL of the previously organized DPPH stock solution and employed for incubation in the shade for 25 min. Eventually, the absorbance of the used samples was noted at 517 nm utilizing UV/Vis spectrophotometry (UV-1800, Shimadzu, Kyoto, Japan). The results were obtained via Equation (3).

% Antioxidant potential = A − B /A *×* 100 (3)

In the equation, A represents control absorbance and B denotes absorbance of the essential oil (EO) and the standard (ascorbic acid).

#### 2.9.2. ABTS Test

The antioxidant capabilities of ELEO were also screened via ABTS bioassay. Around 383 mg of the ABTS and nearly 66.2 mg of K_2_S_2_O_8_ were individually homogenized in the 100 mL analytical-grade methanol and then mixed. Later, around 2 mL from the ABTS solution was carefully incubated with 2 mL of tested samples for 25 min utilizing equal dosages, as explained in the DPPH assay. Moreover, the absorbance of the analyzed samples was estimated at 746 nm utilizing UV/Vis spectrophotometry. The antioxidant ability was analyzed using formula 3.

### 2.10. In Vivo Activities

The in vivo activities were performed using Swiss albino mice with an average weight of 24–30 g bought from the Veterinary Research Institute Peshawar, KP Pakistan. The Swiss albino mice were placed in AWKUM animal’s houses at 20 °C for 45 days following ARRIVE guidelines strictly for the intake of rodent pellets, foodstuff, and water.

#### 2.10.1. Analgesic Assessment

To authenticate the essential oil significance concerning pain therapy, the ELEO of the plant under study was tested following an acetic-acid-stimulated writhing bioassay (AAIWA) using Swiss albino mice as an experimental animals [41]. Furthermore, the experimental animals were equally divided into 5 groups (*n* = 6). The tested samples, including *E. larica* EO, control, and standard, were inoculated very carefully through the intraperitoneal muscle in experimental animals using the sterilized syringe. Around 1 mL of (0.7%) acetic acid (AA) at dosages of 5 mL/kg body weight (BW) were infused into all the groups of the Swiss albino mice. Then, after 45 min, the mice of group 1 (control) and group 2 (standard) were given around 1 mL each of normal saline and aspirin, correspondingly, injected following ARRIVE guidelines. In addition to that, the ELEO was inoculated to the remaining groups of 3rd, 4th, and 5th experimental animals at concentrations of 25, 50, and 100 mg/kg BW dosages, respectively. Furthermore, writhes were calculated to determine the tested samples’ analgesic potential compared to normal saline and analgesic standard for 10 min. The obtained data were represented by % inhibition via Equation (4).

(4) $$\%\;inhibition=A-\frac{B}{A}\times 100\ldots \ldots \ldots \ldots \ldots .$$

where A represents acetic acid induces writhes in Swiss albino mice, and B indicates the analgesic potential of the EO, analgesic standard, and negative control.

#### 2.10.2. Anti-Inflammatory Evaluation

The ELEO was screened to determine its anti-inflammatory effects via carrageenan-induced paw edema assay in the experimental animals, as previously reported by Shah et al. [41]. The Swiss albino mice were properly grouped, as previously stated in the analgesic bioassay. However, inflammation was produced in all the groups of the Swiss albino mice by injecting 1 mL (1%) of carrageenan [44]. Right after 30 min, the Swiss albino mice of group 1 were treated with 1 mL of normal saline (NS) as a negative control, whereas 1 mL of diclofenac sodium (50 mg/kg) was inoculated into group 2 mice following safety measure. The essential oil of EL at dosages of 25, 50, and 100 mg/kg of body weight (BW) were inoculated into the experimental animals of groups 3, 4, and 5, individually. In addition to that, the anti-inflammatory capabilities of examined samples were determined by calculating the Swiss albino mice right paw diameter in the same way as after the 1st, 2nd, and 3rd h, respectively, and resulting data were represented as % inhibition and computed applying Formula (4).

In Formula (4), A represents carrageenan-induced paw edema and B denotes the anti-inflammatory capabilities of essential oil, anti-inflammatory drug (standard), and control (NS) in the diameter of paw edema.

## 3. Statistical Analysis

The data for the in vitro and in vivo screening were taken in triplets and computed via one-way analysis of variance (ANOVA), examined through Bonferroni’s test at significance level *p* = 0.05 (*) and 0.01 (**) via two-way ANOVA. Furthermore, Sidak’s multiple comparisons tests (*p* = (ns > 0.9999, **** < 0.0001)) for statistical validity were performed. Furthermore, the free radical scavenging effects were determined through a nonlinear regression graph plotted amongst % inhibition and dosages of the experimental samples, and the IC_50_ was ascertained utilizing the GraphPad Prism 9.0.1 (2021) software for windows (San Diego, CA, USA, 2020) through the following formula.

Y = 100/1 + (ˆHillSlope)

where 1 represents the inhibitor concentration and denotes the inhibitor’s reaction. HillSlope shows curve steepness.

All experiments were conducted in triplicate to lower the likelihood of errors, and variations in the findings are described as Standard Error of Mean (SEM). The IC_50_ of all analyzed substances was determined utilizing EZ-FIT (Perrella Scientific, Inc., Amherst, MA, USA). The cytotoxic significance was calculated via IBM SPSS Statistics 26 software to examine the dose response and calculation of IC_50_.

## 4. Result and Discussion

### 4.1. Chemical Composition Identification of the ELEO

**Figure 1:**
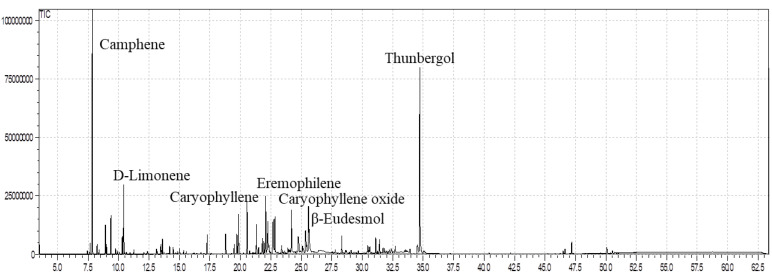
GC-MS analysis of the essential oil of *E. larica*.

In the current context, the GC/MS profile demonstrated that ELEO contained sixty components (60), representing 95.25% of the total oil constituents (Table 1). Camphene (16.41%) and thunbergol (15.33%) were the major components, followed by limonene (4.29%), eremophilene (3.77%), β-caryophyllene (3.47%), β-eudesmol (3.51%), and α-selinene (3.26%) (Table 1; Figure 1). In addition to that, minor constituents noticed in the ELEO were cubebol (2.97%), caryophyllene oxide (2.85%), β-elemene (2.71%), δ-cadinene (2.48%), β-myrcene (2.21%), and τ-cadinol (2.45%). Hydrocarbons such as pentacosane, heptacosane, and nonacosane as well as hydroxy-acids such as 2-methyl-2,2-dimethyl-1,1-(2-hydroxy)-1-propanoic acid and 2-methyl-1,3-hydroxy-2,4,4-trimethylene propanoic acid were identified previously in the wax of *E. larica* [36]. These outcomes agree with those described in previous analyses [45,46,47]. The EOs of *E. caracasana* and *E. cotinifolia* harvested from Mérida, Venezuela, were found to be rich in β-caryophyllene (33.7%), caryophyllene oxide (6.4%), α-selinene (7.6%), α-humulene (18.8%), aromadendrene (8.4%), α-copaene (9.3%), and germacrene-D (21.5%) [45]. A higher amount of β-eudesmol (18.22%), caryophyllene oxide (8.61%), and β-selinene (4.21%) was determined in the *E. fischeriana* essential oil from China [46]. On the other hand, small quantities of camphene (0.98%), β-selinene (0.76%), τ-cadinol (0.93%), caryophyllene oxide (1.74%), and germacrene-D (0.67%) were reported in the EO of *E. mauritanica* belonging to Egypt [47]. Moreover, the EO of *E. mauritanica* was found to be higher in cembrene A (18.66%), verticiol (17.05%), and limonene (7.91%) [47] compared to our results. As Lokar et al. [48] reported, different habitats, seasons, geographical areas, and harvesting periods can affect the quantitative composition and variation of EO in similar plant species from diverse regions. The difference in the quantity of the active ingredients implies the variation in the ecosystem diversity where plant species grow. According to Salehi et al. [49], the EOs obtained from the plant species belonging to the genus *Euphorbia* consist of sesquiterpenes as a leading bioactive constituent in both oxygenated and non-oxygenated forms; however, β-caryophyllene was observed as the prime component up to 7% in the EOs of the genus *Euphorbia* [49]. Furthermore, our results displayed a higher quantity of camphene and thunbergol, utilized as a distinctive indicator of *E. larica* from the southern areas of Oman.

### 4.2. In Vitro Cytotoxicity Potential

The ELEO was screened against the human breast cancer cell line MDA-MB-231 to find out the inhibition of growth in cancer cells from low to high doses and authenticate the literature previously published by Bhalla et al. [13] and Loizzo et al. [50] about the cytotoxic capabilities of plant essential oils. At the same time, human normal breast epithelial cell line MCF-10A was kept as a control in the experiment. The MTT [3-(4,5-dimethylthiazol-2-yl)-2,5-diphenyltetrazolium bromide] bioassay was applied to evaluate the decrease in cancer cell viability persuaded through cytotoxic agents. For MDA-MB-231 and MCF-10A, the IC_50_, % inhibition, and % viability of ELEO are shown in Table 2. The results of the MTT assay revealed that the ELEO was effective against MDA-MB-231 cells with an IC_50_ =183.8 ± 1.6 μg/mL.

To find out whether the cytotoxic effects of ELEO were selective for malignant cells as equated to the non-malignant cells, the non-tumorigenic MCF-10A cells were exposed to the ELEO at dosages of 3, 10, 30, 100, and 300 μg/mL at the same concentration of the cancer cells. The findings revealed that these cells were less liable to the actions of the ELEO, especially at a higher dosage of 300 μg/mL. Furthermore, current outcomes also depicted that the triple-negative MDA-MB-231 cells, having an aggressive phenotype, responded more positively to ELEO and presented considerable cytotoxicity. In addition to that, less cytotoxic capacity was observed when non-tumorigenic MCF-10A cells were screened to the essential oil of the plant under study, indicating that ELEO has the significant potential to act as an effective remedy to cure breast cancer. Furthermore, essential oils also contain monomers which are well known for their cytotoxic significance, as stated by Yang et al. [51] and Maraveas et al. [52].

The current findings reflected that the tested sample offered appreciable capabilities against MDA-MB-231 cells with an IC_50_ value of 183.8 ± 1.6 μg/mL. The cytotoxic potential might be ascribed to the essential oil due to the presence of β-caryophyllene, β-elemene, and α-selinene, which were previously described by Compagnone et al. [53] in the EOs of *Croton micans* and *Croton matourensis* having cytotoxic potential. Thus, our findings favor the data reported by Azaat et al. [54] and Yagi et al. [55] for the essential oils extracted from the genus *Euphorbia.* The similarities observed in the mentioned literature with current data might be due to the essential oils extracted from the same genus and also the same approach used to examine the cytotoxic effects. However, our observed findings are not equated with the results presented by Estanislao et al. [56] and Lahmadi et al. [57] for *Eucalyptus* and *Decatropis bicolor*, respectively. The variation among the effects might be due to the differences in the plant genus and habitat, and the bioactive content in the plants also varies due to the quality and water availability, as reflected in the literature stated by Rawat et al. [58].

### 4.3. α-Glucosidase and Carbonic Anhydrase-II Bioassays

The essential oil of *E. larica* essential oil displayed potent antidiabetic capacity, with an IC_50_ = 9.63 ± 0.22 µg/mL as compared to the available marketed drug acarbose with an IC_50_ = 377.71 ± 1.34 µg/mL. The promising ability attributed to the tested sample is due to the presence of these active compounds such as linalool, limonene, and caryophyllene oxide in the essential oil, as earlier stated in the literature of Najibullah et al. [59], Basak et al. [60], and Jelassi et al. [61]. However, essential oils are affluent sources of monomers that have promising capabilities to act as antidiabetics, as reflected by Bigham et al. [62] in plant species *Teucrium polium* and *Musca domestica* and also revealed by Riyaphan et al. [63].

**Figure 2:**
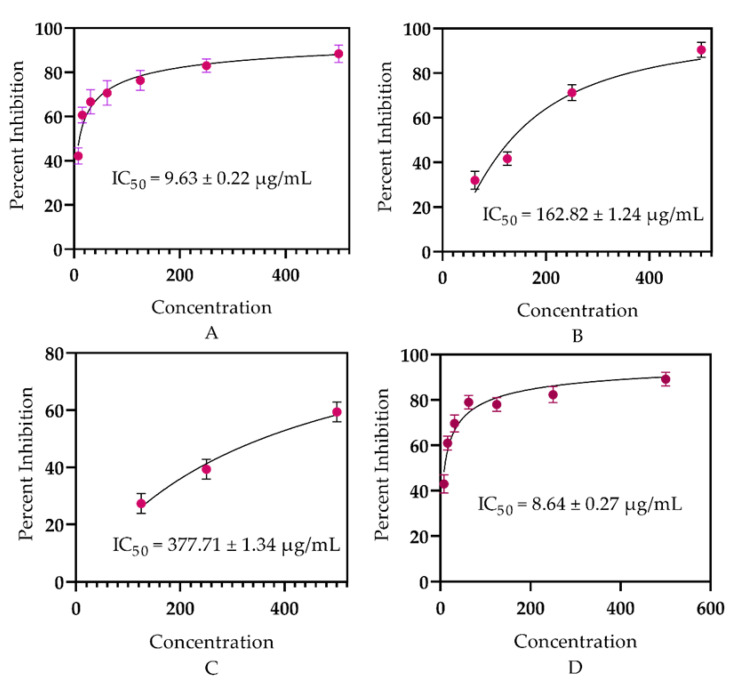
In vitro antidiabetic potential of ELEO: (**A**) α-glucosidase, (**B**) carbonic anhydrase-II, (**C**) acarbose, (**D**) acetazolamide.

Our study authenticates the literature as earlier documented by Valarezo et al. [64] and Yu et al. [65] for the antidiabetic potential of some plant species of the genus *Euphorbia*. However, our data are not matched with the data presented by Salazar et al. [66] for *Origanum vulgare* and Dang et al. [67] for some Vietnamese citrus peel essential oils. The resemblances may perhaps be due to the genus similarities and disparities might be due to the differences in the plant family, as well as habitat, as these factors alter the chemical composition of the constituents within the plants. Furthermore, the same sample (ELEO) was investigated against the CA-II enzyme, displaying above 50% inhibition, and was found to be active against CA-II with an IC_50_ = 162.82 ± 1.24 µg/mL (Figure 2) in comparison with standard acetazolamide (8.64 ± 0.27 µg/mL). These findings revealed that ELEO might be utilized as a therapeutic method for type 2 diabetes and other diseases associated with the CA-II enzyme.

### 4.4. Antimicrobial Potential

#### 4.4.1. Antibacterial Significance

**Figure 3:**
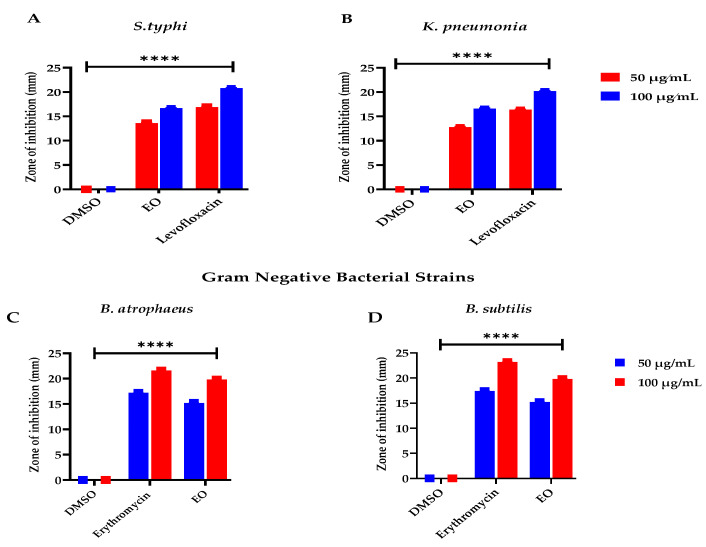
Antibacterial potential of ELEO. levofloxacin and erythromycin were used as standards against the gram-negative and gram-positive bacterial strains, respectively, whereas the DMSO was used as a negative control. (**A**) represents bacterial resistance against *S. typhi,* (**B**) *K. pneumonia*, (**C**) *B. atrophaeus*, and (**D**) *B. subtilis*. All the data was taken in triplicate (*n* = 3) and analyzed through two-way ANOVA, via Tukey’s multiple comparison test, ns = >0.9999, *p* = **** <0.0001.

In recent eras, with the advancement of therapeutic practices in the scientific field, the remedial properties of the plant’s essential oils have drawn great interest due to their fewer adverse effects, appreciable pharmacological implication, and economic practicability, especially to overwhelmed microbial infections, as stated by Chouhan et al. [68]. *E. larica* essential oil presented considerable activity against the tested microbes. However, the EO was found effective against the Gram-positive tested bacteria and offered 19.8 ± 0.02 mm resistance against *B. atrophaeus* followed by *B. subtilis* with ZOI 18.2 ± 0.04 mm, as equated to the Gram-negative bacterial strain *S. typhi* having 15.4 ± 0.02 mm ZOI. At the same time, 15.3 ± 0.07 mm ZOI was observed against *K. pneumonia* (Figure 3). In addition to that, the standard used for the Gram-positive and Gram-negative bacterial strains erythromycin and levofloxacin offered significant antibacterial resistance. The antibacterial feature attributed to the essential oils is due to the presence of α-selinene, as earlier reported by Silva et al. [69] in *Myrcia alagoensis*. The plant effluents in eremophilene and caryophyllene were also reported for their antibacterial capabilities, as revealed in the literature published by Utegenova et al. [70] and Moo et al. [71], which were also examined in the plant under study. In addition to that, essential oils also contain monomers that have antibacterial capabilities, as stated by Su et al. [72] and Elegir et al. [73]. Moreover, our outcomes agreed with the records expressed by Zhu et al. [74], Adedoyin et al. [75], and Olaoluwa et al. [76] regarding *Euphorbia helioscopia, Euphorbia heterophylla,* and *Euphorbia hirta*, as these plant species belong to the same genus and most of the chemical constituents are the same. In addition, the recent data do not match the data revealed by Carovic et al. [77] and Wilkinson et al. [78], as the plant species contain different phytoconstituents due to habitat variability, edaphic and climatic factors, as well as the quality and availability of water.

#### 4.4.2. Antifungal Capabilities

**Figure 4:**
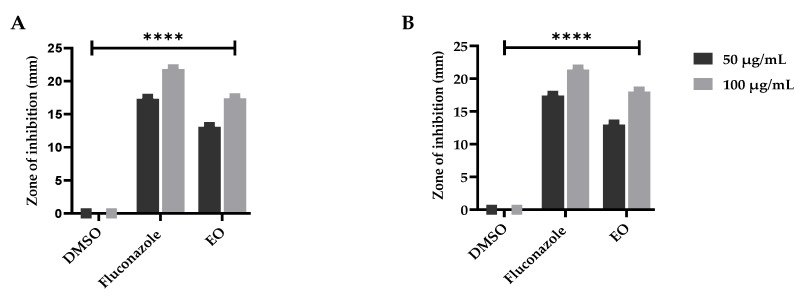
Antifungal potential of the *E. larica* essential oils against (**A**) *A. parasiticus* and (**B**) *A. niger.* DMSO was utilized as a negative control and fluconazole as a standard (positive control). All the data was taken in triplicate (*n* = 3) and analyzed through two-way ANOVA, via Tukey’s multiple comparison test, ns = >0.9999, *p* = **** <0.0001.

The essential oils have multiple biomedical applications containing antifungal capabilities, as confirmed by Nazzaro et al. [79]. To further validate the previous literature as described by Hu et al. [80], the plant essential oil under study was examined for its antifungal significance and presented an appreciable resistance against the tested fungal strains. However, the maximum ZOI was observed against *Aspergillus parasiticus* at 18.03 ± 0.01 µg/mL followed by *Aspergillus niger* with 17.4 ± 0.03 ZOI as compared to the used antifungal standard; however, the negative control was examined as inactive (Figure 4). The significant resistance against the fungal strains is attributed to the presence of β-eudesmol, as earlier reported by Ho et al. [81], and camphene and thunbergol, as previously documented by Angioni et al. [82] and Mitic et al. [83], respectively. The role of monomers acting as an antifungal agents cannot be denied, which was earlier documented by Chen et al. [84] and Neves et al. [85].

Thus, our current findings agree with the data depicted by Rao et al. [86] and Mahmoud et al. [87], however, our results deviate from those as stated by Bansod et al. [88], and Oumzil et al. [89]. Different plant species vary in their chemical constituents, which can overcome various human health complications, as well as that the environmental gradients influence the bioactive constituents within a plant species, as earlier stated by Shah et al. [90].

### 4.5. Antioxidant Capability

**Figure 5:**
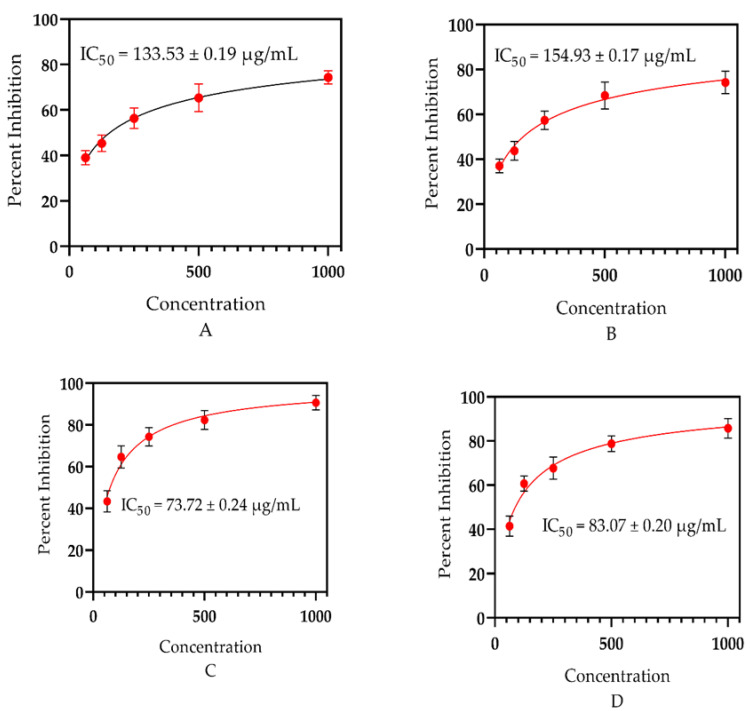
(**A**) Antioxidant significance of ELEO using DPPH assay, (**B**) antioxidant capability of *E. larica* essential oil via ABTS assay, (**C**) antioxidant effect of the standard ascorbic acid via DPPH, and (**D**) antioxidant activity of the standard via ABTS assay.

Essential oils are a promising basis for neutralizing free radicals, as earlier documented by Amorati et al. [91] and Shah et al. [41]. To confirm this further, the essential oils obtained from the whole plant of *E. larica* Boiss. (Euphorbiaceae) were investigated for their free radical scavenging capacities. The assessment revealed that the analyzed samples had considerable potential to scavenge the free radicals (Figure 5). However, *E. larica* essential oil was observed with utmost significance via DPPH assay with an IC50 = 133.53 ± 0.19 µg/mL, as equated to the ABTS assay having an IC50 = 154.93 ± 0.17 µg/mL. In addition to that, the standard ascorbic acid offered an IC50 = 73.72 ± 0.24 and IC50 = 83.07 ± 0.20 µg/mL via DPPH and ABTS assay, respectively. The appreciable ability attributed to ELEO is due to the presence of α-selinene, eremophilene, and caryophyllene in a high amounts, which was heretofore explained by Chandra et al. [92] and Ahmadvand et al. [93] in *Callicarpa macrophylla* and *Artemisia persica*, respectively. However, monomers also can neutralize free radicals, as documented previously by Maraveas et al. [52] and Su et al. [72].

Our current findings consented with the data stated by Elshamy et al. [94] and Essa et al. [47] for the essential oils of *E. heterophylla* and *E. mauritanica* L., as the plant species under study belongs to the same genus, having the majority of common bioactive constituents which can neutralize the free radicals. However, our data did not match with the study documented by Jie et al. [46], and the plant studied, *Euphorbia fischeriana*, belongs to the same genus collected from China. The chemical ingredients vary among the plants of the same genus due to their habitat, quality, and availability of water. In addition to that, plant species from a different genus, *Ochradenus*, presented similar antioxidant potential due to the same method of essential oil extraction, habitat, and a similar approach used to examine the free radical scavenging effect, as revealed in the literature of Ullah et al. [37].

### 4.6. Anti-Inflammatory Significance

The essential oil of *E. larica* was examined to highlight the anti-inflammatory capabilities induced via carrageenan in Swiss albino mice. The essential oil presented significant potential in the reduction in paw edema from low to high doses at 55.40%, 58.78%, and 65.54%, respectively, as equated to the standard with 73.64% inhibition (Table 3). At the same time, the normal saline was observed to be inactive (Table 4). Our current findings authenticate the data confirmed by Miguel et al. [4] for EOs. The anti-inflammatory significance depicted by the plant essential oils, mainly caryophyllene oxide, were previously reported by Chao et al. [95] in *Cinnamomum osmophloeum* and β-caryophyllene and observed in the essential oils of *Syzygium cumini* and *Psidium guajava*, having promising anti-inflammatory activity, as stated by Siani et al. [96]. Furthermore, our study is supported by the details mentioned in the data explained by Sinan et al. [97] and De-Morais et al. [98] in the genus *Euphorbia.* The similarities in the aforementioned plants were due to the plant species from the same genus because of the common bioactive constituents and the extraction method of essential oils. Moreover, our current data do not match with the literature documented by Adeosun et al. [99], Souza et al. [100], and Aboluwodi et al. [101]. The variations observed might be due to the plant belonging to a different genus, environmental factors, collection time, and quality and availability of water, as well that the pollutants in the water contents also have adverse effects on the bioactive constituents, as explained by Shah et al. [102].

### 4.7. Analgesic Capabilities

The exploration of natural and green products is continuously expanding the use of EOs, and thus pressure is exerted on these materials from emerging nations. It is estimated that the requirement for EOs over the globe will increase by 7.5% from 2020–2027 due to their diverse medical applications, including analgesic agents, as documented by Scuteri et al. [103]. To confirm the previous study, the essential oils of *E. larica* were examined to emphasize the capabilities to relieve pain induced in Swiss albino mice via acetic acid. The screening revealed that the *E. larica* essential oil presented significant potential of 36.95, 48.18, and 58.33% reduction in writhes caused by the acetic acid in the Swiss albino mice from low to high doses of 25, 50, and 100 mg/kg, correspondingly (Table 4). However, the normal saline was observed to be inactive, and the analgesic standard (aspirin) depicted 68.47% inhibition. The analgesic significance ascribed to the plant essential oil under study is due to the presence of limonene, as previously expressed by Guimaraes et al. [104], and caryophyllene, as elaborated by Bakir et al. [105]. Thus, our results consented with the data earlier reported by Ahmad et al. [106] and Majid et al. [107] for *Euphorbia decipiens* and *Euphorbia dracunculoides,* respectively. The similarities in our results were observed because the plant under investigation belonged to the same genus, *Euphorbia*, and the approach used to examine the analgesic activity was also the same. In addition to that, our recent results did not agree with the data presented by Kaskoos et al. [108] for *Lippia citriodora* and *Citrus limon* or the data reported in the literature stated for *Cyperus rotundus* by Chen et al. [109]. The variation in the data of our findings with the previously documented literature is due to the variation among the plant species, as well as that the different plant species possess various active constituents which are influenced by various aspects, such as habitat, climatic and edaphic factors, and water availability, as stated by Aboukhalid et al. [110].

## 5. Conclusions

From the current study, it is assumed that ELEO comprises promising bioactive constituents having multiple biomedical features. For the first time, we reported the GC-MS profiling and in vitro and in vivo pharmacological assessment of ELEO. The ELEO exhibited significant antimicrobial activity, antidiabetic and antiproliferative effects, the capability to scavenge free radicals, the potential to cure inflammation, and a significant effect on pain. The GC-MS analysis of the oil presented 60 bioactive compounds that contributed to 95.25% of ingredients with dominant volatile constituents, i.e., camphene (16.41%), thunbergol (15.33%), limonene (4.29%), eremophilene (3.77%), and β-eudesmol (3.51%). The ELEO attributed an appreciable analgesic significance (58.33%) and an anti-inflammatory potential (65.54%). The oil displayed prominent antioxidant activity using DPPH (IC_50_ 133.53 ± 0.19 µg/mL) and ABTS (IC_50_ of 154.93 ± 0.17 µg/mL) assays. The EO determined effective resistance of 19.8 ± 0.02 mm ZOI against *B. atrophaeus* (Gram-positive bacteria) and *Aspergillus parasiticus* (18.03 ± 0.01 µg/mL, fungal strain). Moreover, the ELEO displayed significant cytotoxic potential with an IC_50_ = 183.8 ± 1.6 μg/mL against the triple-negative breast cancer (MDA-MB-231) cell lines and did not produce much harm to the normal cell lines (MCF-10A). Furthermore, significant inhibition was observed against α-glucosidase and carbonic anhydrase-II enzymes. In addition to that, further analyses are proposed to identify and isolate the responsible bioactive constituents for the investigated complications.

## Figures and Tables

**Table 1:** GC-MS assessment of the essential oil of *E. larica*.

Compound’s Name	Rt (min)	Rel. Conc. (%)	KI ^(a)^	KI ^(b)^	Identification ^(c)^
3-Thujene	7.65	0.62	928	935	KI, MS
Camphene	7.85	16.41	935	942	KI, MS
2,4-Thujadiene	8.39	0.26	957	963	KI, MS
Sabinene	8.91	1.68	964	972	KI, MS
Laevo-β-pinene	9.00	0.59	965	975	KI, MS
β-Myrcene	9.37	2.21	981	999	KI, MS
α-Phellandrene	9.75	0.39	997	1013	KI, MS
3-Carene	9.92	0.21	1005	1019	KI, MS
p-Cymene	10.30	1.04	1011	1033	KI, MS
Limonene	10.42	4.29	1018	1037	KI, MS
γ-Terpinene	11.25	0.28	1047	1067	KI, MS
Linalool	12.34	0.17	1082	1106	KI, MS
Perillen	12.40	0.17	1086	1108	KI, MS
α-Campholenal	13.10	0.38	1102	1134	KI, MS
2,9-Dimethyl-5-decyne	13.17	0.17	1103	1136	KI, MS
L-Pinocarveol	13.46	0.67	1143	1147	KI, MS
cis-Verbenol	13.52	0.21	1110	1149	KI, MS
trans-Verbenol	13.61	0.94	1128	1153	KI, MS
p-Mentha-1,5-dien-8-ol	14.18	0.48	1148	1174	KI, MS
Terpinen-4-ol	14.48	0.42	1175	1185	KI, MS
Myrtenol	15.00	0.41	1174	1204	KI, MS
Levoverbenone	15.34	0.23	1191	1218	KI, MS
cis-Carveol	15.54	0.14	1208	1226	KI, MS
Bornyl acetate	17.26	1.24	1269	1292	KI, MS
α-Terpinyl acetate	18.79	1.53	1322	1355	KI, MS
Copaene	19.48	0.63	1376	1383	KI, MS
β-Bourbonene	19.71	1.31	1386	1393	KI, MS
β-Elemene	19.84	2.71	1398	1398	KI, MS
β-Caryophyllene	20.54	3.47	1421	1428	KI, MS
Humulene	21.32	1.89	1454	1462	KI, MS
Alloaromadendrene	21.49	0.49	1459	1469	KI, MS
γ-Muurolene	21.80	1.02	1471	1483	KI, MS
Germacrene D	21.93	0.78	1480	1489	KI, MS
Eremophilene	22.07	3.77	1486	1494	KI, MS
δ-Selinene	22.14	0.17	1509	1497	KI, MS
α-Selinene	22.26	3.26	1500	1503	KI, MS
Cubebol	22.68	2.97	1512	1522	KI, MS
δ-Cadinene	22.83	2.48	1514	1529	KI, MS
Elemol	23.39	0.65	1535	1554	KI, MS
Germacrene D-4-ol	23.99	0.21	1570	1582	KI, MS
Caryophyllene oxide	24.19	2.85	1575	1591	KI, MS
Viridiflorol	24.61	0.18	1594	1611	KI, MS
Humulene 1,2-epoxide	24.74	1.18	1596	1617	KI, MS
γ-Eudesmol	24.79	0.71	1627	1620	KI, MS
Cubenol	25.09	0.46	1631	1634	KI, MS
τ-Cadinol	25.34	2.45	1637	1646	KI, MS
δ-Cadinol	25.45	0.56	1646	1651	KI, MS
β-Eudesmol	25.58	3.51	1648	1656	KI, MS
Benzyl Benzoate	27.77	0.21	1765	1767	KI, MS
α-Phellandrene, dimer	28.32	1.11	1801	1795	KI, MS
m-Camphorene	31.10	1.06	1960	1945	KI, MS
Cembrene A	31.42	0.95	1970	1963	KI, MS
p-Camphorene	31.70	0.29	1977	1979	KI, MS
geranyl-α-terpinene	32.22	0.16	2001	2008	KI, MS
Verticiol	32.70	0.43	2036	2036	KI, MS
Cembrenol	34.52	1.43	2061	2140	KI, MS
Thunbergol	34.73	15.33	2073	2157	KI, MS
24-Norursa-3,9(11),12-triene	46.48	0.23	3042	2999	KI, MS
24-Noroleana-3,12-diene	46.64	0.33	3057	3013	KI, MS
24-Norursa-3,12-diene	47.19	0.87	3105	3060	KI, MS
Total identified compounds (%)	95.25				

^(a)^ KI_Lit_: published Kovats retention indices; ^(b)^ KI_obs_: experimental Kovats index relative to C8–C28 *n*-alkanes; Rt: retention time; Rel. Conc. (%): relative concentration (%); ^(c)^ the identification of ELEO compounds based on the comparison of the mass spectral data and Kovats indices (KI) with those of NIST Mass Spectral Library (2011).

**Table 2:** Cytotoxic significance of ELEO.

Cell Line Used	Conc (μg/mL)	% Viability	% Inhibition	IC_50_ (μg/mL)
MDA-MB-231	3	96.57	3.42	183.8 ± 1.6
	10	88.37	11.62	
	30	79.82	20.17	
	100	61.76	38.23	
	300	35.24	64.75	
MCF-10A	3	97.40	2.59	>300
	10	91.04	8.95	
	30	86.16	13.83	
	100	83.99	16.08	
	300	81.24	18.75	

**Table 3:** Anti-inflammatory capabilities of ELEO.

		Changes in Paw Diameter Experimental Animals (Mean ± SEM)				
Tested Sample	Dosage	1 h	2 h	3 h	Aver. Paw Diameter	% Inhibition
Carrageenan	1 mL	1.18 ± 0.06	1.46 ± 0.02	1.81 ± 0.02	1.48 ± 0.01	---
Normal saline	1 mL	1.15 ± 0.05	1.43 ± 0.02	1.77 ± 0.05	1.45 ± 0.03	---
Diclofenac sodium	50 mg/kg	0.47 ± 0.03	0.41 ± 0.04	0.30 ± 0.02	0.39 ± 0.06	73.64
EO	25	0.75 ± 0.04	0.66 ± 0.03	0.59 ± 0.04	0.66 ± 0.02 *	55.40
	50	0.71 ± 0.02	0.62 ± 0.04	0.51 ± 0.03	0.61 ± 0.05 *	58.78
	100	0.57 ± 0.05	0.51 ± 0.03	0.45 ± 0.05	0.51 ± 0.04 *	65.54

Carrageenan inducer of paw edema, normal saline as a negative control, and diclofenac sodium was used as a standard, EO essential oil with *p* ≤ 0.05 *.

**Table 4:** Analgesic capabilities of ELEO.

Tested Samples	Dosage Used	Counted Writhes’ Average (Mean *±* SEM)	Percentage in Writhes’ Reduction
Acetic acid	1 mL	27.6 ± 0.04	
Normal saline	1 mL	27.4 ± 0.06	
Aspirin	1 mL	8.7 ± 0.05	68.47
EO	25 mg/kg	17.4 ± 0.04 **	36.95
	50	14.3 ± 0.06 **	48.18
	100	11.5 ± 0.03 **	58.33

Acetic acid as inducer, normal saline used as a negative control, standard aspirin EO essential oil, *p* = 0.01 denoted as **.

## Data Availability

The data presented in this study are available in the article.
